# From Insight to Eyesight: Unveiling the Secrets of the Insulin-Like Growth Factor Axis in Retinal Health

**DOI:** 10.14336/AD.2024.0128

**Published:** 2024-10-01

**Authors:** Raju V. S. Rajala, Ammaji Rajala

**Affiliations:** ^1^Departments of Ophthalmology, University of Oklahoma Health Sciences Center, Oklahoma, USA.; ^2^Physiology, University of Oklahoma Health Sciences Center, Oklahoma, USA.; ^3^Cell Biology, University of Oklahoma Health Sciences Center, Oklahoma, USA.; ^4^Dean McGee Eye Institute, Oklahoma, Oklahoma, USA.

**Keywords:** Insulin-like growth factor, retina, retinal pigment epithelium, photoreceptor cells, age-related macular degeneration, aging

## Abstract

Insulin-like growth factor-1 (IGF-1) plays a diverse role in the retina, exerting its effects in both normal and diseased conditions. Deficiency of IGF-1 in humans leads to issues such as microcephaly, mental retardation, deafness, and postnatal growth failure. IGF-1 is produced in the retinal pigment epithelium (RPE) and activates the IGF-1 receptor (IGF-1R) in photoreceptor cells. When IGF-1R is absent in rod cells, it results in the degeneration of photoreceptors, emphasizing the neuroprotective function of IGF signaling in these cells. Contrastingly, in neovascular age-related macular degeneration (AMD), there is an overexpression of both IGF-1 and IGF-1R in RPE. The mechanisms behind this altered regulation of IGF-1 in diseased states are currently unknown. This comprehensive review provides recent insights into the role of IGF-1 in the health and disease of the retina, raising several unanswered questions that still need further investigation.

## 1. Introduction

**Figure 1.**
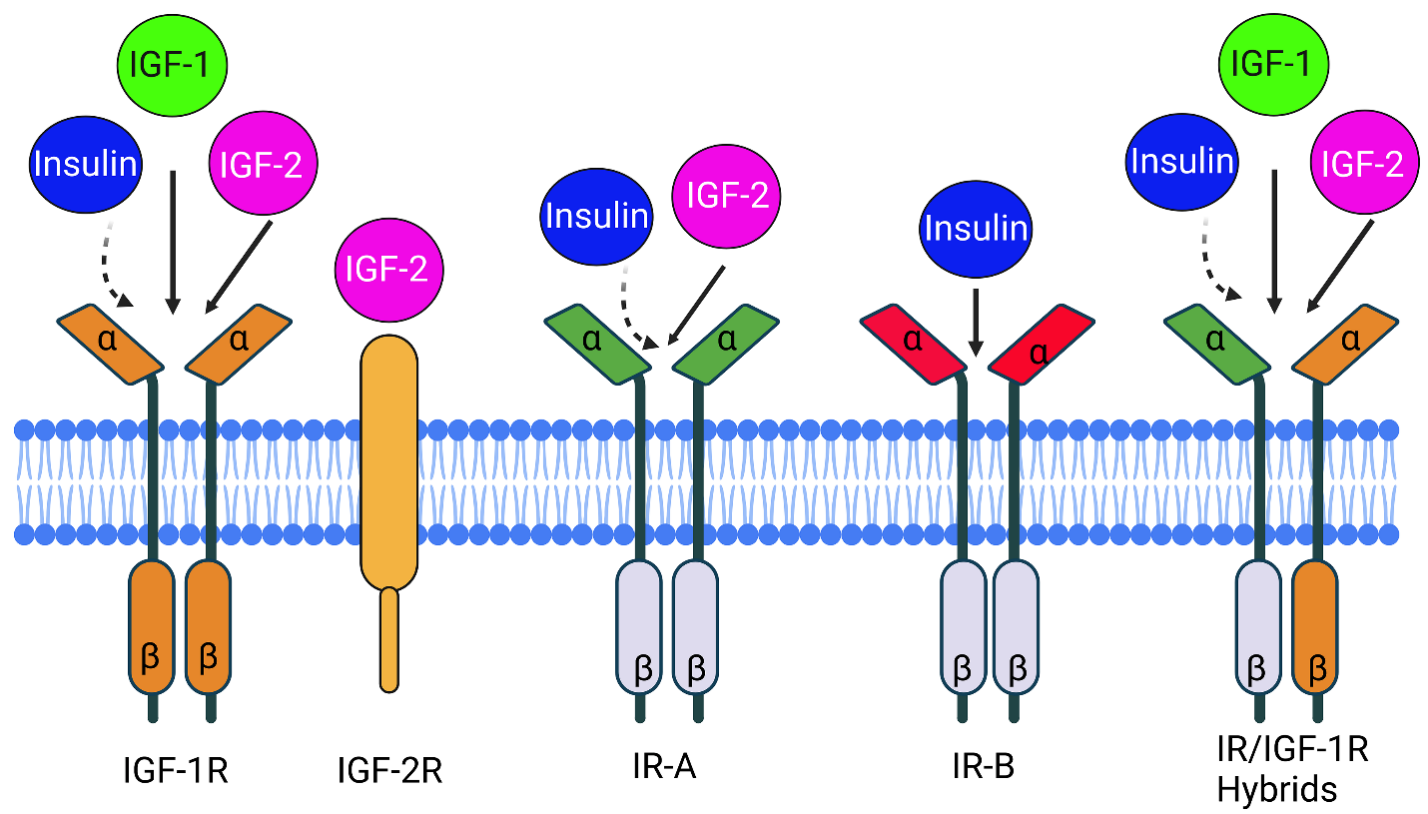
The insulin/insulin-like growth factors (IGF) system involves the interaction between ligands and receptors. The receptors, insulin receptor (IR) and insulin-like growth factor receptor (IGF-1R) consist of two αβ dimers each. IR has two splice variants, IR-A and IR-B, with differing extracellular α subunits. IR αβ dimers can bind to IGF-1R αβ dimers, creating hybrid receptors called IR/IGF-1R. On the other hand, the IGF-2R receptor, lacking intrinsic tyrosine kinase activity, is unrelated to IR and IGF-1R. The solid arrows indicate high affinity, while broken arrows indicate low affinity, representing the relative binding affinities of each receptor to insulin, IGF-1, and IGF-2. This figure was created with BioRender.com.

**Figure 2.**
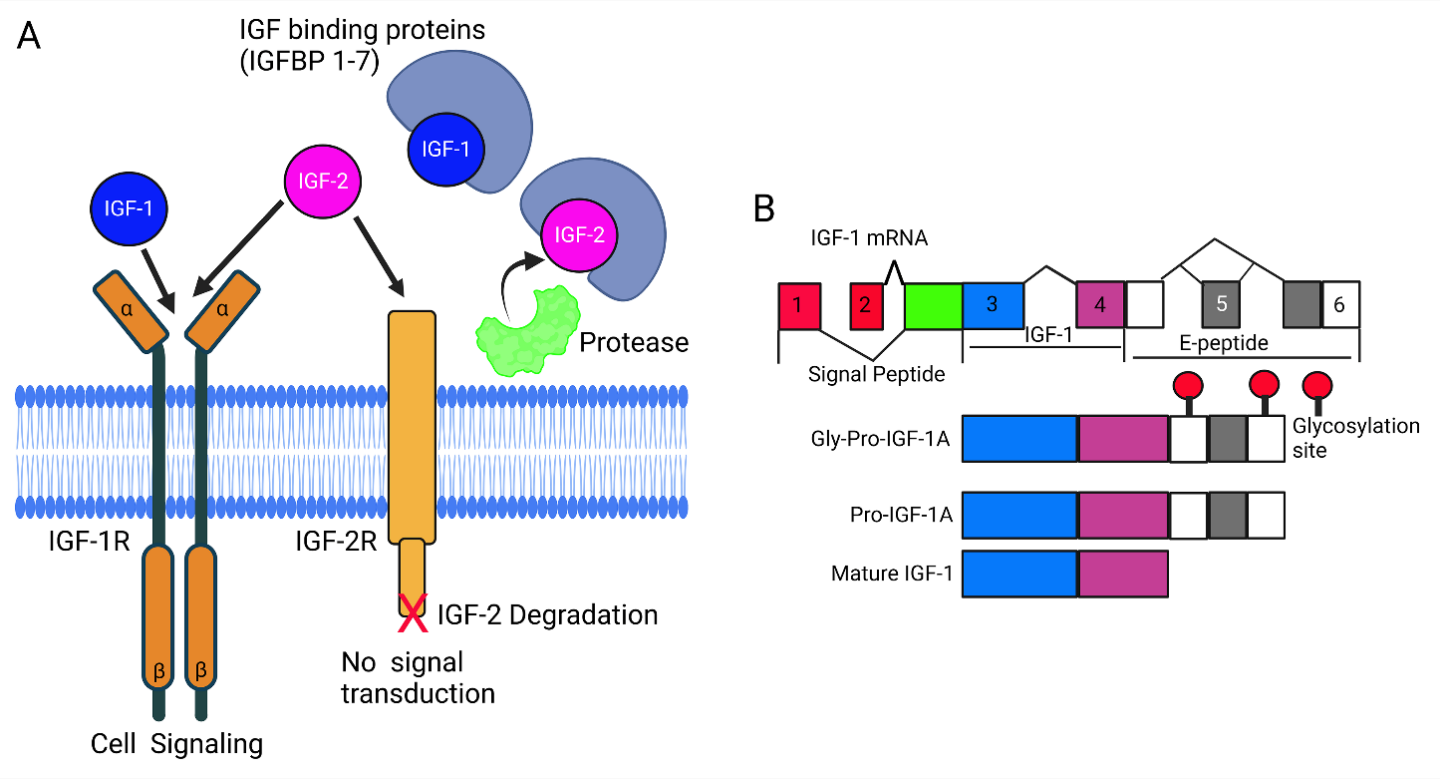
The interplay between insulin-like growth factor (IGF) and insulin-like growth factor binding proteins (IGFBPs). Interactions between IGF and IGFBPs can reduce free IGF levels, decreasing insulin-like growth factor receptor (IGF-1R) activation and inhibiting cellular response. IGF-2R, without tyrosine kinase activity, counteracts the impact of IGF-2 by binding to and internalizing this signaling molecule. Subsequently, the internalized ligand undergoes lysosomal degradation. Associations of IGFBPs with extracellular matrix (ECM) components can reduce the affinity of IGFBP for IGFs, thereby releasing bioactive IGF. The actions of proteases can also increase free IGF levels since IGFBP fragments have reduced affinity for IGFs. IGFBPs may have direct effects mediated by as-yet uncharacterized IGFBP receptors (A). Schematic presentation of alternative splicing of the *Igf1* gene (B). This figure was created with BioRender.com.

The insulin-like growth factor (IGF) family consists of ligands, namely IGF-1, IGF-2, and insulin, along with receptors, including IGF-1R, M6P/IGF-2R, and insulin receptor (IR) (Fig.1). Additionally, IGF-binding proteins (IGFBP-1-7) are integral components that play crucial roles in both normal human physiology and various disease states (Fig.2) [1]. In this commentary, we will focus on examining the fundamental components of the IGF system, exploring their functions in both normal physiology and significant pathological conditions. We have described several animal models that have played a crucial role in enhancing our understanding of the IGF system and its regulation [2].

IGF-1 exerts its effects both systemically through the bloodstream and locally within tissues, regulating cellular growth, differentiation, and survival [2]. This, in turn, governs overall body growth [2]. During prenatal stages, IGF-2 levels peak, influencing crucial aspects of growth [3]. In adults, IGF-2 assumes tissue-specific roles, particularly in maintaining stem cell populations [3]. Despite its close relation to the insulin receptor (IR), IGF-1R has distinct physiological functions at both the cell surface and within the nucleus [1]. On the other hand, M6P/IGF-2R acts differently, serving as a scavenger by facilitating the internalization and degradation of IGF-2 [1]. The IGFBPs, binding to IGF-1 and IGF-2 in the circulation, extend their half-lives and modulate tissue access, thereby regulating IGF function (Fig.2A). Additionally, IGFBPs exhibit cell effects independent of the IGF ligand [1].

## 2. IGF-1: A Key Player in Growth Regulation, Genetic Complexity, and Neurotrophic Functions

IGF-1 is primarily produced in the liver in response to Growth Hormone (GH) stimulation [4]. The production of GH begins in the hypothalamus, where Growth Hormone-Releasing Hormone (GHRH) stimulates the pituitary gland to release GH [4]. Research indicates that the secretion of GH decreases as individuals age [5]. When GH binds to its receptors on liver cells, it triggers the synthesis and release of IGF-1 [4]. Additionally, IGF-1 can be produced locally in various tissues, exerting both autocrine and paracrine effects. IGF-1 plays a central role in mediating many of the growth-promoting effects of GH, contributing to cellular growth, differentiation, and metabolic regulation [6]. The interaction between GH and IGF-1 forms a coordinated system that regulates growth and development throughout the body [4].

IGF-1 is derived from the *Igf1* gene, exhibiting over 90% conservation across species [7]. The gene can undergo alternative splicing at both the 5’- and 3’-ends, producing multiple isoforms (Fig. 2B) [7, 8]. Irrespective of the transcribed isoform, a pre-pro-peptide is translated, comprising a signal peptide for secretion, the mature IGF-1 peptide, and a C-terminal extension known as the E-peptide [9]. Following signal peptide cleavage, pro-IGF-1 (mature IGF-1 plus an E-peptide) may undergo additional processing before secretion [9]. This includes intracellular protease-mediated cleavage of the E-peptide to release mature IGF-1 [9], maintenance of pro-IGF-1 for secretion without cleavage [10-12], or N-glycosylation in the E-peptide of the predominant IGF-1 isoform (IGF-1A), followed by secretion (referred to as *Gly-Pro-IGF-1*) [13]. Consequently, three forms of IGF-1 protein could potentially exist in the extracellular milieu: mature IGF-1, nonglycosylated pro-IGF-1, and glycosylated pro-IGF-1(Fig.2B). Therefore, it is crucial to comprehend not only the quantity but also the proportion of IGF-1 forms produced to assess the true biological activity of this growth factor.

IGF-1, also known as somatomedin C, is a hormone with a molecular structure similar to insulin [14]. It plays a crucial role in childhood growth and has anabolic effects in adults. The IGF-1 protein, encoded by the IGF1 gene in humans, consists of 70 amino acids arranged in a single chain with three intramolecular disulfide bridges [15]. It has a molecular weight of 7,649 daltons [15]. Previous studies have found that polymorphisms in the IGF-1 gene are associated with conditions such as breast cancer [16], gastric cancer [17], coronary artery disease [18], childhood IgA nephritis [19, 20], diabetic retinopathy [21, 22], AMD [23] and high-grade myopia [24].

The aging brain has been observed to experience a decline in IGF-1 levels [25]. Homozygous mutations in the IGF-1 gene lead to IGF-1 deficiency in humans, resulting in microcephaly, mental retardation, deafness, and postnatal growth failure [26, 27]. This underscores the crucial role of IGF-1 as a neurotrophic factor [26, 27]. Additionally, global IGF-1 knockout mice exhibit age-related vision loss and congenital deafness [28]. Due to these characteristics, IGF-1 has been utilized to counteract neurodegeneration in experimental animal models of brain injury [29] and retinal neurodegeneration [30-33].

## 3. Roles of Insulin, Insulin-Like Growth Factor-1 (IGF-1), and Insulin-Like Growth Factor-2 (IGF-2) in the Retina

In the retina, both IR [34, 35], and IGF-1R [36] receptors are expressed besides the existence of IR/IGF-1R hybrid receptors [37]. Insulin receptors exist as two isoforms, type A and type B stem from alternative splicing, specifically the exclusion of exon 11 to generate a type A receptor [38, 39]. In contrast, in the liver, the inclusion of this exon results in a type B receptor [38, 39]. The retina mainly expresses the type A IR isoform which has a high basal kinase activity comparable to that in the liver of well-fed animals [37, 40]. However, unlike the liver, this activity remains stable and does not vary with feeding and fasting [37]. Notably, it declines rapidly after the onset of insulin-deficient diabetes but can be restored through local and systemic administration of insulin [41]. Following this, it has been shown that basal IR kinase activity is regulated by IGF-2 [42]. This conclusion stems from elegant experiments involving neutralizing antibodies against IGF-2, which, when introduced, resulted in a decrease in IR kinase activity in the retinas of normal rats [42]. Specifically reducing IGF-2 in the bloodstream led to decreased IR phosphorylation in retinal cells [42]. In adults, the mRNA content of IGF-2 in the retina was ten times higher than in young rats and significantly exceeded levels found in the liver [42]. In the context of diabetes, IGF-2 mRNA levels in the retina decreased, while IGF-1 or IR mRNA levels remained unaffected [42]. Diabetes also caused reduced levels of IGF-2 and IGF-1 in the vitreous fluid [42]. Notably, directly administering IGF-2 into the eye (both mature and pro-forms) increased IR and Akt kinase activities in the retinas of diabetic rats [42]. These findings underscore the role of IGF-2 as the primary signaling molecule that regulates basal IR activity in the retina. Consistent with these studies, an earlier study indicates that IGF-1R, insulin receptor (IR), and the ligand IGF-2 are expressed almost exclusively in photoreceptors and blood vessels [43].

Recently, research has demonstrated that phagocytosis in the retina plays a role in promoting local insulin production within the eye [44]. Additionally, the deletion of the *Ins2* gene, either globally or specifically in the retinal pigment epithelium (RPE), leads to reduced retinal glucose uptake in starved male mice [44]. This deletion also disrupts retinal physiology, induces defects in phototransduction, and worsens photoreceptor loss in a mouse model of retinitis pigmentosa [44]. Together, this information highlights RPE cells as a source of locally induced insulin in the retina through phagocytosis, suggesting their capacity to impact retinal physiology and contribute to disease processes [44]. Furthermore, in the retina, IR activation is light-dependent and regulated through a non-canonical rhodopsin-regulated pathway independent of circulatory insulin [45]. Reducing IGF-2 [42] or insulin production in the RPE [44] doesn't completely eliminate insulin receptor (IR) kinase activity in the retina. This implies that light-dependent IR kinase activity functions independently of ligands.

IGF-1R serves as a receptor tyrosine kinase responsible for carrying out the functions of IGF-1, which it binds to with a strong affinity [25]. On the other hand, IGF-2 and insulin exhibit a lower affinity when binding to this receptor [25]. Unlike IR, there was no light-dependent increased activity of IGF-1R in the retina [46], however, light-stress-induced activation of IGF-1R in the retina and photoreceptor cells resulted in the activation of phosphoinositide 3-kinase (PI3K)/Akt [46]. Both IR and IGF-1R are expressed in photoreceptor cells, and rod-specific deletion of IR has no phenotype under normal physiological conditions, however, light stress causes photoreceptor degeneration [47]. Interestingly, rod-specific deletion of IGF-1R resulted in age-related photoreceptor degeneration without stress [48]. These studies underscore the neuroprotective role of IGF-1R in photoreceptor cells during normal physiological conditions.

The mechanism through which retinal IGF-1R is activated by tissue- or cell-specific IGF-1 production remained unknown until recently. Even though the liver is the main site for IGF-1 production [49], our recent study showed that deleting the IGF1 gene in the liver, leading to the absence of circulating IGF-1, does not affect retinal function [48]. An earlier study shows that IGF-1 is expressed throughout the retina but at much lower levels [43]. This suggests that IGF-1 may be locally produced in the retina, with observed expression in Müller glial cells [50]. Interestingly, the loss of IGF-1 in Müller cells did not influence retinal function [48]. Previous studies have indicated the presence of IGF-1 immunoreactivity in the inner photoreceptor matrix (IPM), positioned between the RPE and photoreceptor cells [51], which also contains high levels of IGF-1 binding protein (IGFBP) regulating IGF-1 availability [51]. Cultured human RPE cells have been shown to synthesize and release IGF-1, proposing the RPE as a potential source of IPM-IGF-1 *in vivo* [51]. The coexistence of IGF-1 and IGFBP in the IPM, coupled with IGF-1 receptors on both photoreceptor and RPE cells, suggests the existence of an autocrine-paracrine system in the outer retina. Our recent study supports this notion by revealing elevated IGF-1 and IGFBP3 protein expression in the RPE and increased IGF-1R expression in photoreceptor cells [48]. Notably, our findings align with a prior study demonstrating over 100-fold higher IGF-1R expression compared to insulin receptors in photoreceptor cells [43]. Together, these results imply that the RPE might be a source of IGF-1 for the activation of photoreceptor IGF-1R. Additional functional studies are necessary to confirm that the RPE is the primary source of IGF-1 production in the retina.

## 4. The Relationship between IGF-1 and Age-Related Macular Degeneration and Diabetic Retinopathy

Mice lacking IGF-1 exhibit age-related vision loss and congenital deafness [52] and loss of IGF-1R in rods results in age-related photoreceptor degeneration [48]. The neuroprotective role of IGF-1 in the progression of retinitis pigmentosa has been demonstrated [53]. Previous research revealed that IGF-1 had a beneficial impact by reducing reactive gliosis and apoptosis in *ex vivo* retinal explants from rd10 mice [53]. Importantly, the efficacy of IGF-1 in preserving photoreceptor viability was compromised when retinal microglia were eliminated using clodronate-filled liposomes [53]. Similarly, IGF-1 could not suppress reactive Müller gliosis in the absence of microglial cells. Overall, these findings suggest that the microglia mediate, at least in part, the beneficial effects of IGF-1 [53]. The results highlight the crucial role of cross-talk between microglia and Müller glial cells in neuroprotection [53]. Additionally, the study indicates that microglia is essential for the neuroprotective effects of IGF-1 in the dystrophic retina [53].

There is abundant evidence supporting the link between IGF-1 and processes related to diabetic complications, particularly retinopathy [54-56]. Some studies indicate no correlation between the serum levels of IGF-1 and diabetic retinopathy [57, 58]. However, higher local concentrations of IGF-1 in the vitreous fluid of diabetic patients have been observed [59-62]. This suggests that the local concentration of IGF-1 in tissues is more crucial than circulating IGF-1. In certain tissues, IGF-1 seems to function more as a paracrine factor rather than an endocrine factor.

Moreover, inhibiting abnormal IGF-1 signaling in the retina has been demonstrated to prevent and reverse diabetic retinopathy in male rats [63]. This reversal may be attributed to the synergistic collaboration of IGF-1 with dopamine in mitigating diabetic retinopathy by downregulating VEGF [64]. The evidence suggests that in the early stages of diabetic retinopathy, IGF-1 plays a direct role in regulating both VEGF levels and the disruption of the blood-retina barrier (BRB) [56]. Consistent with earlier studies, transgenic mice with elevated retinal IGF-1 exhibit key features of diabetic eye disease, characterized by increased VEGF expression, ultimately resulting in neovascularization and BRB breakdown [65]. The precise cause of elevated IGF-1 levels in the retina due to diabetes is not completely understood at this time. Despite this, research indicates a correlation between both low and high levels of IGF-I, impaired glucose tolerance, and an increased susceptibility to type 2 diabetes [66]. Further investigation is needed to thoroughly understand the biological mechanisms underlying this intricate phenomenon in the future.

Furthermore, the PI3K pathway and VEGF play crucial roles in various cellular processes, particularly in angiogenesis and cell survival. Both PI3K and VEGF are key regulators of angiogenesis, the process of new blood vessel formation [67]. VEGF stimulates angiogenesis by binding to its receptors on endothelial cells, leading to the activation of the PI3K/Akt signaling pathway [67, 68]. This activation promotes endothelial cell survival, proliferation, and migration, contributing to the formation of new blood vessels [67]. Dysregulation of PI3K and VEGF signaling has been implicated in pathological angiogenesis, such as in cancer and retinal diseases [69]. In cancer, abnormal activation of PI3K and overexpression of VEGF can lead to the formation of new blood vessels to supply nutrients to the tumor [70]. Therapies targeting both PI3K and VEGF pathways are being explored for anti-angiogenic and anti-cancer strategies [71]. In diseases like AMD and diabetic retinopathy, where abnormal angiogenesis can lead to vision impairment, targeting both PI3K and VEGF pathways has been explored as a therapeutic strategy [72]. Inhibitors of VEGF, such as anti-VEGF drugs, are commonly used in clinical practice, and there is ongoing research on the development of PI3K pathway inhibitors. Understanding the crosstalk between PI3K and VEGF pathways is crucial for developing targeted therapies. Combining inhibitors that target both pathways may have synergistic effects in controlling angiogenesis and treating diseases associated with abnormal blood vessel formation [73]. The combined actions of PI3K and VEGF are integral to processes like angiogenesis and cell survival. Their dysregulation is implicated in various diseases, and therapeutic strategies targeting both pathways are being explored for clinical applications.

Neurons and retinal pigment epithelium (RPE) produce IGF-1, yet its targets and effects in choroidal neovascularization (CNV) remain unclear. IGF-1 was abundant in human CNV tissues, and RPE cells had receptors for IGF-1 [74]. When RPE cells from CNV tissues were exposed to IGF-1, there was a consistent rise in intracellular free Ca^2+^ [74]. In unstimulated RPE cell cultures from CNV tissues, VEGF levels in the medium increased steadily over time, and this doubled when the cells were stimulated with IGF-1 [74]. This indicates that IGF-1 in RPE cells activates Ca^2+^ and enhances VEGF secretion, playing a role in the development of neovascularization [74]. Another study reported elevated levels of insulin-like growth factor binding protein 2 (IGFBP-2) and IGF-1 in eyes affected by exudative AMD [75]. This suggests that abnormalities in the expression of IGF-related molecules may play a role in the pathogenesis of exudative AMD [75].

IGF-1 plays a crucial role as a growth factor in the process of retinal angiogenesis [43]. Clinical observations indicate a correlation between low systemic levels of IGF-1 and the severity of retinopathy of prematurity, and IGF-1 is implicated in diabetes [43]. Both IR and IGF-1R in retinal vascular endothelial cells are associated with pathological angiogenesis [43]. Interestingly, mice lacking IR or IGF-1R in endothelial cells experience regular vascular development but show protection against hypoxia-induced retinal neovascularization [76]. However, the complete absence of IGF-1R in the neural retina and blood vessels does inhibit normal retinal vascular development, indicating that IGF-1R in the neural retina plays a role in influencing vascular development [77]. Previous observations have indicated the expression of matrix metallopeptidase 2 (MMP2) and IGF-1R in Muller cells during oxygen-induced retinopathy [78]. The authors concluded that the IGF-1/IGF-1R system plays a role in regulating the levels of active MMP-2 in Müller glial cells, thereby contributing to the remodeling of Müller glial cells during the retinal neovascular process [78]. In another independent study, it was found that the expression of IGF-binding proteins, IGFBP3 and IGFBP5 increases in neovascular tufts when compared to normal vessels [43].

Inflammation is linked to AMD, and the IGF axis may play a role in influencing AMD risk by regulating inflammatory responses [79]. Evidence suggests that intraocular IGF-1, as opposed to systemic IGF-1, is capable of initiating processes that lead to the breakdown of the blood-retinal barrier and an increase in retinal vascular permeability [80]. In eyes with neovascular AMD, there is an elevation of IGF-1 in the ocular fluid of the eye [75]. The IGF-1/IGF-IR system has been associated with anti-inflammatory properties in the central nervous system (CNS) across various pathologies, including type 1 diabetes mellitus [79]. This is achieved by mitigating the inflammatory environment initiated by microglial activation in the hypothalamus [81]. Nonetheless, conflicting effects of IGF-1 have been reported, particularly concerning its proinflammatory impact in different pathological contexts. A recent study conducted in zebrafish has provided evidence that IGF-1 and insulin, along with cytokines, activate shared signaling pathways essential for the reprogramming of Müller glial cells and subsequent retinal regeneration following injury [82]. Furthermore, recent research provides support for the connection between type 2 diabetes mellitus (T2DM) and neurodegeneration through two key factors: the increase in proinflammatory cytokines and the onset of insulin/IGF-1 resistance [79]. In the context of T2DM, proinflammatory cytokines may be responsible for disrupting insulin/IGF-1 signaling pathways in peripheral tissues and the pancreas [83]. Additionally, the accumulation of peripheral proinflammatory mediators, some of which can penetrate the blood-brain barrier, is likely to initiate insulin/IGF-1 resistance in the central nervous system (CNS) [79]. This resistance results in the attenuation of neuroprotective signaling pathways, contributing to the onset of neurodegenerative diseases [53]. The accumulated proinflammatory mediators can also breach the blood-retina barrier and cause retinal insulin/IGF-1 resistance. Additional comprehensive studies are necessary.

## 5. Unanswered Key Questions in the Field of Retinal Studies

**Question 1**. Under normal physiological conditions, research suggests that IGF-1 is produced in the RPE and activates IGF-1R in photoreceptor cells through paracrine signaling. The RPE expresses minimal levels of IGF-1R, and photoreceptors themselves do not generate IGF-1. In the case of AMD, there is an observed increase in the levels of both IGF-1 and IGF-1R in the RPE, leading to an augmented secretion of VEGF. The higher levels of IGF-1R in the RPE may sequester IGF-1, impeding its delivery to photoreceptor cells. As a result, this interference affects the neuroprotective signaling mediated by IGF-1R, thereby affecting the overall neuroprotection provided by the activation of IGF-1R. It is important to note that this hypothesis requires further testing for validation.

**Question 2**. IGF-1 is produced in the retinal pigment epithelium (RPE), and in age-related macular degeneration (AMD), there is an increased expression of IGF-1R. Understanding the genetic factors or transcriptional regulators that activate IGF-1R expression holds significant informational value. Targeting IGF-1R in the RPE has the potential to alleviate the progression of AMD.

**Question 3**. In AMD, there is an elevated expression of insulin-like growth factor binding proteins (IGFBPs) in the RPE [84]. This increased expression may sequester IGF-1, thereby restricting the availability of IGF-1 for photoreceptor cells. Targeting IGFBP(s) in the RPE could redirect IGF-1 to photoreceptors, promoting their survival.

**Question 4**. Typically, growth hormone stimulates the liver to produce IGF-1. However, in the retina, RPE cells produce IGF-1, and it is uncertain whether this production is still regulated by GH. Assessing GH levels in retinal health and disease could provide valuable information.

**Question 5**. Presently, there are no studies on the processing of the *Igf1* gene in the retina, particularly in the RPE. It is essential to explore the gene products in both normal retinal physiology and pathology to gain a comprehensive understanding.

## 6. Conclusions

In this review, we explored the roles of the IGF axis in both general health and retinal diseases. Initially, we highlighted earlier studies that demonstrated the presence of IGF-1, IGF-2, and insulin in the retina. These ligands activate IR and IGF-1R, exerting a neuroprotective effect on the retina under normal physiological conditions. Next, we delved into the underlying pathophysiological connections between IGF-1 and AMD. Lastly, we addressed the existing gaps in knowledge within the field, aiming to uncover how the IGF axis promotes both protective and destructive signals in both health and disease. By addressing these aspects, this review aims to assist readers in designing specific experiments to better comprehend how IGF-1 signals are altered in retinal diseases.

## References

[1] LeRoith D, Holly JMP, Forbes BE (2021) Insulin-like growth factors: Ligands, binding proteins, and receptors. Mol Metab, 52:101245. 33962049 10.1016/j.molmet.2021.101245 PMC8513159

[2] Yakar S, Adamo ML (2012) Insulin-like growth factor 1 physiology: lessons from mouse models. Endocrinol Metab Clin North Am, 41:231-47, v. 22682628 10.1016/j.ecl.2012.04.008 PMC5488279

[3] Ziegler AN, Feng Q, Chidambaram S, Testai JM, Kumari E, Rothbard DE, et al. (2019) Insulin-like Growth Factor II: An Essential Adult Stem Cell Niche Constituent in Brain and Intestine. Stem Cell Reports, 12:816-30. 30905741 10.1016/j.stemcr.2019.02.011 PMC6450461

[4] LeRoith D, Yakar S (2007) Mechanisms of disease: metabolic effects of growth hormone and insulin-like growth factor 1. Nat Clin Pract Endocrinol Metab, 3:302-10. 17315038 10.1038/ncpendmet0427

[5] Hage C, Salvatori R (2023) Growth Hormone and Aging. Endocrinol Metab Clin North Am, 52:245-57. 36948778 10.1016/j.ecl.2022.10.003

[6] Burgner JW 2nd, Ray WJ Jr, (1984) On the origin of the lactate dehydrogenase induced rate effect. Biochemistry, 23:3636-48. 6477889 10.1021/bi00311a010

[7] Barton ER (2006) The ABCs of IGF-I isoforms: impact on muscle hypertrophy and implications for repair. Appl Physiol Nutr Metab, 31:791-7. 17213901 10.1139/h06-054

[8] Durzyńska J, Philippou A, Brisson BK, Nguyen-McCarty M, Barton ER (2013) The pro-forms of insulin-like growth factor I (IGF-I) are predominant in skeletal muscle and alter IGF-I receptor activation. Endocrinology, 154:1215-24. 23407451 10.1210/en.2012-1992 PMC3578996

[9] Duguay SJ, Milewski WM, Young BD, Nakayama K, Steiner DF (1997) Processing of wild-type and mutant proinsulin-like growth factor-IA by subtilisin-related proprotein convertases. J Biol Chem, 272:6663-70. 9045697 10.1074/jbc.272.10.6663

[10] Conover CA, Baker BK, Bale LK, Clarkson JT, Liu F, Hintz RL (1993) Human hepatoma cells synthesize and secrete insulin-like growth factor Ia prohormone under growth hormone control. Regul Pept, 48:1-8. 10.1016/0167-0115(93)90330-b 8265808

[11] Conover CA, Baker BK, Hintz RL (1989) Cultured human fibroblasts secrete insulin-like growth factor IA prohormone. J Clin Endocrinol Metab, 69:25-30. 2732297 10.1210/jcem-69-1-25

[12] Wilson HE, Westwood M, White A, Clayton PE (2001) Monoclonal antibodies to the carboxy-terminal Ea sequence of pro-insulin-like growth factor-IA (proIGF-IA) recognize proIGF-IA secreted by IM9 B-lymphocytes. Growth Horm IGF Res, 11:10-7. 11437469 10.1054/ghir.2000.0182

[13] Bach MA, Roberts CT Jr, Smith EP, LeRoith D (1990) Alternative splicing produces messenger RNAs encoding insulin-like growth factor-I prohormones that are differentially glycosylated in vitro. Mol Endocrinol, 4:899-904. 2233747 10.1210/mend-4-6-899

[14] Höppener JW, de Pagter-Holthuizen P, Geurts van Kessel AH, Jansen M, Kittur SD, Antonarakis SE, et al. (1985) The human gene encoding insulin-like growth factor I is located on chromosome 12. Hum Genet, 69:157-60. 2982726 10.1007/BF00293288

[15] Rinderknecht E, Humbel RE (1978) The amino acid sequence of human insulin-like growth factor I and its structural homology with proinsulin. J Biol Chem, 253:2769-76. 632300

[16] Wang Q, Liu L, Li H, Tao P, Qi Y, Li J (2016) Effects of High-Order Interactions among IGFBP-3 Genetic Polymorphisms, Body Mass Index and Soy Isoflavone Intake on Breast Cancer Susceptibility. PLoS One, 11:e0162970. 27631779 10.1371/journal.pone.0162970 PMC5024997

[17] Oh SY, Shin A, Kim SG, Hwang JA, Hong SH, Lee YS, et al. (2016) Relationship between insulin-like growth factor axis gene polymorphisms and clinical outcome in advanced gastric cancer patients treated with FOLFOX. Oncotarget, 7:31204-14. 27144430 10.18632/oncotarget.9100 PMC5058750

[18] Ricketts SL, Rensing KL, Holly JM, Chen L, Young EH, Luben R, et al. (2011) Prospective study of insulin-like growth factor-I, insulin-like growth factor-binding protein 3, genetic variants in the IGF1 and IGFBP3 genes and risk of coronary artery disease. Int J Mol Epidemiol Genet, 2:261-85. 21915365 PMC3166154

[19] Hahn WH, Suh JS, Cho BS (2011) Polymorphisms of insulin-like growth factor-1 (IGF-1) and IGF-1 receptor (IGF-1R) contribute to pathologic progression in childhood IgA nephropathy. Growth Factors, 29:8-13. 21047277 10.3109/08977194.2010.532126

[20] Wei L, Fu R, Liu X, Wang L, Wang M, Yu Q, et al. (2018) Rs1520220 and Rs2195239 Polymorphisms of IGF-1 Gene Associated with Histopathological Grades in IgA Nephropathy in Northwestern Chinese Han Population. Kidney Blood Press Res, 43:80-7. 29402846 10.1159/000486914

[21] Zhang J, Chen X, Zhang L, Peng Y (2017) IGF1 gene polymorphisms associated with diabetic retinopathy risk in Chinese Han population. Oncotarget, 8:88034-42. 29152139 10.18632/oncotarget.21366 PMC5675691

[22] Uthra S, Raman R, Mukesh BN, Rajkumar SA, Kumari RP, Agarwal S, et al. (2007) Diabetic retinopathy and IGF-1 gene polymorphic cytosine-adenine repeats in a Southern Indian cohort. Ophthalmic Res, 39:294-9. 17851271 10.1159/000108124

[23] Chiu CJ, Conley YP, Gorin MB, Gensler G, Lai CQ, Shang F, et al. (2011) Associations between genetic polymorphisms of insulin-like growth factor axis genes and risk for age-related macular degeneration. Invest Ophthalmol Vis Sci, 52:9099-107. 22058336 10.1167/iovs.11-7782 PMC3231967

[24] Metlapally R, Ki CS, Li YJ, Tran-Viet KN, Abbott D, Malecaze F, et al. (2010) Genetic association of insulin-like growth factor-1 polymorphisms with high-grade myopia in an international family cohort. Invest Ophthalmol Vis Sci, 51:4476-9. 20435602 10.1167/iovs.09-4912 PMC2941166

[25] Korschen HG, Illing M, Seifert R, Sesti F, Williams A, Gotzes S, et al. (1995) A 240 kDa protein represents the complete beta subunit of the cyclic nucleotide-gated channel from rod photoreceptor. Neuron, 15:627-36. 7546742 10.1016/0896-6273(95)90151-5

[26] Bonapace G, Concolino D, Formicola S, Strisciuglio P. (2003) A novel mutation in a patient with insulin-like growth factor 1 (IGF1) deficiency. J Med Genet, 40:913-7. 14684690 10.1136/jmg.40.12.913 PMC1735341

[27] Woods KA, Camacho-Hubner C, Savage MO, Clark AJ (1996) Intrauterine growth retardation and postnatal growth failure associated with deletion of the insulin-like growth factor I gene. N Engl J Med, 335:1363-7. 8857020 10.1056/NEJM199610313351805

[28] Rodriguez-de la Rosa L, Fernandez-Sanchez L, Germain F, Murillo-Cuesta S, Varela-Nieto I, de la Villa P, et al. (2012) Age-related functional and structural retinal modifications in the Igf1-/- null mouse. Neurobiol Dis, 46:476-85. 22402333 10.1016/j.nbd.2012.02.013

[29] Saatman KE, Contreras PC, Smith DH, Raghupathi R, McDermott KL, Fernandez SC, et al. (1997) Insulin-like growth factor-1 (IGF-1) improves both neurological motor and cognitive outcome following experimental brain injury. Exp Neurol, 147:418-27. 9344566 10.1006/exnr.1997.6629

[30] KermerP, KlockerN, LabesM, BahrM (2000) Insulin-like growth factor-I protects axotomized rat retinal ganglion cells from secondary death via PI3-K-dependent Akt phosphorylation and inhibition of caspase-3 In vivo. J Neurosci, 20:2-8.10632601

[31] Dupraz S, Grassi D, Karnas D, Nieto Guil AF, Hicks D, Quiroga S (2013) The insulin-like growth factor 1 receptor is essential for axonal regeneration in adult central nervous system neurons. PLoS One, 8:e54462. 23349896 10.1371/journal.pone.0054462 PMC3548777

[32] Liegl R, Löfqvist C, Hellström A, Smith LE. (2016) IGF-1 in retinopathy of prematurity, a CNS neurovascular disease. Early Hum Dev, 102:13-9. 27650433 10.1016/j.earlhumdev.2016.09.008 PMC5085844

[33] Politi LE, Rotstein NP, Salvador G, Giusto NM, Insua MF (2001) Insulin-like growth factor-I is a potential trophic factor for amacrine cells. J Neurochem, 76:1199-211. 11181839 10.1046/j.1471-4159.2001.00128.x

[34] Waldbillig RJ, Fletcher RT, Chader GJ, Rajagopalan S, Rodrigues M, LeRoith D (1987) Retinal insulin receptors. 1. Structural heterogeneity and functional characterization. Exp Eye Res, 45:823-35. 3123267 10.1016/s0014-4835(87)80099-4

[35] Waldbillig RJ, Fletcher RT, Chader GJ, Rajagopalan S, Rodrigues M, LeRoith D (1987) Retinal insulin receptors. 2. Characterization and insulin-induced tyrosine kinase activity in bovine retinal rod outer segments. Exp Eye Res, 45:837-44. 3322853 10.1016/s0014-4835(87)80100-8

[36] Waldbillig RJ, Fletcher RT, Somers RL, Chader GJ (1988) IGF-I receptors in the bovine neural retina: structure, kinase activity and comparison with retinal insulin receptors. Exp Eye Res, 47:587-607. 2972556 10.1016/0014-4835(88)90097-8

[37] Reiter CE, Sandirasegarane L, Wolpert EB, Klinger M, Simpson IA, Barber AJ, et al. (2003) Characterization of insulin signaling in rat retina in vivo and ex vivo. Am J Physiol EndocrinolMetab, 285:E763-E74. 10.1152/ajpendo.00507.2002 12799319

[38] Gosbell AD, Favilla I, Baxter KM, Jablonski P (2000) Insulin receptor and insulin receptor substrate-I in rat retinae. Clin ExperimentOphthalmol, 28:212-5. 10.1046/j.1442-9071.2000.00305.x 10981802

[39] Seino S, Bell GI (1989) Alternative splicing of human insulin receptor messenger RNA. Biochem Biophys ResCommun, 159:312-6. 10.1016/0006-291x(89)92439-x 2538124

[40] Rajala RV, Wiskur B, Tanito M, Callegan M, Rajala A (2009) Diabetes reduces autophosphorylation of retinal insulin receptor and increases protein-tyrosine phosphatase-1B activity. Invest Ophthalmol Vis Sci, 50:1033-40. 19029027 10.1167/iovs.08-2851 PMC2694133

[41] Reiter CE, Wu X, Sandirasegarane L, Nakamura M, Gilbert KA, Singh RS, et al. (2006) Diabetes reduces basal retinal insulin receptor signaling: reversal with systemic and local insulin. Diabetes, 55:1148-56. 16567541 10.2337/diabetes.55.04.06.db05-0744

[42] Zolov SN, Imai H, Losiewicz MK, Singh RSJ, Fort PE, Gardner TW (2021) Insulin-like growth factor-2 regulates basal retinal insulin receptor activity. J Biol Chem, 296:100712. 33915127 10.1016/j.jbc.2021.100712 PMC8138762

[43] Lofqvist C, Willett KL, Aspegren O, Smith AC, Aderman CM, Connor KM, et al. (2009) Quantification and localization of the IGF/insulin system expression in retinal blood vessels and neurons during oxygen-induced retinopathy in mice. Invest Ophthalmol Vis Sci, 50:1831-7. 18997086 10.1167/iovs.08-2903

[44] Iker Etchegaray J, Kelley S, Penberthy K, Karvelyte L, Nagasaka Y, Gasperino S, et al. (2023) Phagocytosis in the retina promotes local insulin production in the eye. Nat Metab, 5:207-18. 36732622 10.1038/s42255-022-00728-0 PMC10457724

[45] Rajala RV, McClellan ME, Ash JD, Anderson RE (2002) In vivo regulation of phosphoinositide 3-kinase in retina through light-induced tyrosine phosphorylation of the insulin receptor beta-subunit. J BiolChem, 277:43319-26. 10.1074/jbc.M206355200 12213821

[46] Dilly AK, Rajala RV (2008) Insulin growth factor 1 receptor/PI3K/AKT survival pathway in outer segment membranes of rod photoreceptors. Invest Ophthalmol Vis Sci, 49:4765-73. 18566464 10.1167/iovs.08-2286 PMC2670430

[47] Rajala A, Tanito M, Le YZ, Kahn CR, Rajala RV (2008) Loss of neuroprotective survival signal in mice lacking insulin receptor gene in rod photoreceptor cells. J BiolChem, 283:19781-92. 10.1074/jbc.M802374200 PMC2443670 18480052

[48] Rajala A, Teel K, Bhat MA, Batushansky A, Griffin TM, Purcell L, et al. (2022) Insulin-like growth factor 1 receptor mediates photoreceptor neuroprotection. Cell Death Dis, 13:613. 35840554 10.1038/s41419-022-05074-3 PMC9287313

[49] Ohlsson C, Mohan S, Sjögren K, Tivesten A, Isgaard J, Isaksson O, et al. (2009) The role of liver-derived insulin-like growth factor-I. Endocr Rev, 30:494-535. 19589948 10.1210/er.2009-0010 PMC2759708

[50] Fu S, Dong S, Zhu M, Sherry DM, Wang C, You Z, et al. (2015) Muller Glia Are a Major Cellular Source of Survival Signals for Retinal Neurons in Diabetes. Diabetes, 64:3554-63. 26068541 10.2337/db15-0180 PMC4587642

[51] Waldbillig RJ, Pfeffer BA, Schoen TJ, Adler AA, Shen-Orr Z, Scavo L, et al. (1991) Evidence for an insulin-like growth factor autocrine-paracrine system in the retinal photoreceptor-pigment epithelial cell complex. J Neurochem, 57:1522-33. 1717648 10.1111/j.1471-4159.1991.tb06347.x

[52] Nassar N, Horn G, Herrmann C, Scherer A, McCormick F, Wittinghofer A (1995) The 2.2 A crystal structure of the Ras-binding domain of the serine/threonine kinase c-Raf1 in complex with Rap1A and a GTP analogue. Nature, 375:554-60. 7791872 10.1038/375554a0

[53] Arroba AI, Alvarez-Lindo N, van Rooijen N, de la Rosa EJ (2011) Microglia-mediated IGF-I neuroprotection in the rd10 mouse model of retinitis pigmentosa. Invest Ophthalmol Vis Sci, 52:9124-30. 22039242 10.1167/iovs.11-7736

[54] Merimee T (1997) The interface between diabetic retinopathy, diabetes management, and insulin-like growth factors. J Clin Endocrinol Metab, 82:2806-8. 9284700 10.1210/jcem.82.9.4265

[55] Janssen JA, Jacobs ML, Derkx FH, Weber RF, van der Lely AJ, Lamberts SW (1997) Free and total insulin-like growth factor I (IGF-I), IGF-binding protein-1 (IGFBP-1), and IGFBP-3 and their relationships to the presence of diabetic retinopathy and glomerular hyperfiltration in insulin-dependent diabetes mellitus. J Clin Endocrinol Metab, 82:2809-15. 9284701 10.1210/jcem.82.9.4180

[56] Poulaki V, Joussen AM, Mitsiades N, Mitsiades CS, Iliaki EF, Adamis AP (2004) Insulin-like growth factor-I plays a pathogenetic role in diabetic retinopathy. Am J Pathol, 165:457-69. 15277220 10.1016/S0002-9440(10)63311-1 PMC1618554

[57] Payne JF, Tangpricha V, Cleveland J, Lynn MJ, Ray R, Srivastava SK (2011) Serum insulin-like growth factor-I in diabetic retinopathy. Mol Vis, 17:2318-24. 21921983 PMC3171491

[58] Hyer SL, Sharp PS, Brooks RA, Burrin JM, Kohner EM. (1988) Serum IGF-1 concentration in diabetic retinopathy. Diabet Med, 5:356-60. 2968886 10.1111/j.1464-5491.1988.tb01005.x

[59] Merimee TJ, Zapf J, Froesch ER (1983) Insulin-like growth factors. Studies in diabetics with and without retinopathy. N Engl J Med, 309:527-30. 6348545 10.1056/NEJM198309013090904

[60] Grant M, Russell B, Fitzgerald C, Merimee TJ (1986) Insulin-like growth factors in vitreous. Studies in control and diabetic subjects with neovascularization. Diabetes, 35:416-20. 2420665 10.2337/diab.35.4.416

[61] Inokuchi N, Ikeda T, Imamura Y, Sotozono C, Kinoshita S, Uchihori Y, et al. (2001) Vitreous levels of insulin-like growth factor-I in patients with proliferative diabetic retinopathy. Curr Eye Res, 23:368-71. 11910526 10.1076/ceyr.23.5.368.5441

[62] Burgos R, Mateo C, Cantón A, Hernández C, Mesa J, Simó R (2000) Vitreous levels of IGF-I, IGF binding protein 1, and IGF binding protein 3 in proliferative diabetic retinopathy: a case-control study. Diabetes Care, 23:80-3. 10857973 10.2337/diacare.23.1.80

[63] Xi G, Wai C, Clemmons D (2019) Inhibition of Aberrant IGF-I Signaling in Diabetic Male Rat Retina Prevents and Reverses Changes of Diabetic Retinopathy. J Diabetes Res, 2019:6456032. 31049357 10.1155/2019/6456032 PMC6458945

[64] Upreti S, Sen S, Nag TC, Ghosh MP (2022) Insulin like growth factor-1 works synergistically with dopamine to attenuate diabetic retinopathy by downregulating vascular endothelial growth factor. Biomed Pharmacother, 149:112868. 35378500 10.1016/j.biopha.2022.112868

[65] Ruberte J, Ayuso E, Navarro M, Carretero A, Nacher V, Haurigot V, et al. (2004) Increased ocular levels of IGF-1 in transgenic mice lead to diabetes-like eye disease. J Clin Invest, 113:1149-57. 15085194 10.1172/JCI19478 PMC385397

[66] Friedrich N, Thuesen B, Jørgensen T, Juul A, Spielhagen C, Wallaschofksi H, et al. (2012) The association between IGF-I and insulin resistance: a general population study in Danish adults. Diabetes Care, 35:768-73. 22374641 10.2337/dc11-1833 PMC3308317

[67] Graupera M, Potente M (2013) Regulation of angiogenesis by PI3K signaling networks. Exp Cell Res, 319:1348-55. 23500680 10.1016/j.yexcr.2013.02.021

[68] Ferrara N, Gerber HP, LeCouter J (2003) The biology of VEGF and its receptors. Nat Med, 9:669-76. 12778165 10.1038/nm0603-669

[69] Goel S, Duda DG, Xu L, Munn LL, Boucher Y, Fukumura D, et al. (2011) Normalization of the vasculature for treatment of cancer and other diseases. Physiol Rev, 91:1071-121. 21742796 10.1152/physrev.00038.2010 PMC3258432

[70] Lugano R, Ramachandran M, Dimberg A (2020) Tumor angiogenesis: causes, consequences, challenges and opportunities. Cell Mol Life Sci, 77:1745-70. 31690961 10.1007/s00018-019-03351-7 PMC7190605

[71] Carmeliet P, Jain RK (2011) Molecular mechanisms and clinical applications of angiogenesis. Nature, 473:298-307. 21593862 10.1038/nature10144 PMC4049445

[72] Penn JS, Madan A, Caldwell RB, Bartoli M, Caldwell RW, Hartnett ME (2008) Vascular endothelial growth factor in eye disease. Prog Retin Eye Res, 27:331-71. 18653375 10.1016/j.preteyeres.2008.05.001 PMC3682685

[73] Patel SA, Nilsson MB, Le X, Cascone T, Jain RK, Heymach JV. (2023) Molecular Mechanisms and Future Implications of VEGF/VEGFR in Cancer Therapy. Clin Cancer Res, 29:30-9. 35969170 10.1158/1078-0432.CCR-22-1366 PMC10274152

[74] Rosenthal R, Wohlleben H, Malek G, Schlichting L, Thieme H, Bowes RC, et al. (2004) Insulin-like growth factor-1 contributes to neovascularization in age-related macular degeneration. Biochem Biophys Res Commun, 323:1203-8. 15451424 10.1016/j.bbrc.2004.08.219

[75] Cha DM, Woo SJ, Kim HJ, Lee C, Park KH (2013) Comparative analysis of aqueous humor cytokine levels between patients with exudative age-related macular degeneration and normal controls. Invest Ophthalmol Vis Sci, 54:7038-44. 24106111 10.1167/iovs.13-12730

[76] Kondo T, Vicent D, Suzuma K, Yanagisawa M, King GL, Holzenberger M, et al. (2003) Knockout of insulin and IGF-1 receptors on vascular endothelial cells protects against retinal neovascularization. J Clin Invest, 111:1835-42. 12813019 10.1172/JCI17455 PMC161423

[77] Hellström A, Carlsson B, Niklasson A, Segnestam K, Boguszewski M, de Lacerda L, et al. (2002) IGF-I is critical for normal vascularization of the human retina. J Clin Endocrinol Metab, 87:3413-6. 12107259 10.1210/jcem.87.7.8629

[78] Lorenc VE, Subirada Caldarone PV, Paz MC, Ferrer DG, Luna JD, Chiabrando GA, et al. (2018) IGF-1R Regulates the Extracellular Level of Active MMP-2, Pathological Neovascularization, and Functionality in Retinas of OIR Mouse Model. Mol Neurobiol, 55:1123-35. 28097474 10.1007/s12035-017-0386-9

[79] Arroba AI, Campos-Caro A, Aguilar-Diosdado M, Valverde ÁM (2018) IGF-1, Inflammation and Retinal Degeneration: A Close Network. Front Aging Neurosci, 10:203. 30026694 10.3389/fnagi.2018.00203 PMC6041402

[80] Haurigot V, Villacampa P, Ribera A, Llombart C, Bosch A, Nacher V, et al. (2009) Increased intraocular insulin-like growth factor-I triggers blood-retinal barrier breakdown. J Biol Chem, 284:22961-9. 19473988 10.1074/jbc.M109.014787 PMC2755703

[81] Zhang G, Yu L, Chen ZY, Zhu JS, Hua R, Qin X, et al. (2016) Activation of corticotropin-releasing factor neurons and microglia in paraventricular nucleus precipitates visceral hypersensitivity induced by colorectal distension in rats. Brain Behav Immun, 55:93-104. 26743854 10.1016/j.bbi.2015.12.022

[82] Wan J, Zhao XF, Vojtek A, Goldman D (2014) Retinal injury, growth factors, and cytokines converge on β-catenin and pStat3 signaling to stimulate retina regeneration. Cell Rep, 9:285-97. 25263555 10.1016/j.celrep.2014.08.048 PMC4194164

[83] Fève B, Bastard JP (2009) The role of interleukins in insulin resistance and type 2 diabetes mellitus. Nat Rev Endocrinol, 5:305-11. 19399017 10.1038/nrendo.2009.62

[84] Kim EJ, Grant GR, Bowman AS, Haider N, Gudiseva HV, Chavali VRM (2018) Complete Transcriptome Profiling of Normal and Age-Related Macular Degeneration Eye Tissues Reveals Dysregulation of Anti-Sense Transcription. Sci Rep, 8:3040. 29445097 10.1038/s41598-018-21104-7 PMC5813239

